# The impact of *Rumex vesicarius* seed water extracts on mice fertility

**DOI:** 10.1007/s11356-021-16335-7

**Published:** 2021-09-18

**Authors:** Ahmad Rashed Alhimaidi, Aiman Abdullah. Ammari, Mohammad Khair Okla, Muath Qasem Algadi, Ramzi Ahmed Amran, Hissah Ibrahim Alhusayni, Mohammed Ahmad Alhimaidi

**Affiliations:** 1 Zoology Department, College of Science, King Saud University, PO Box 2455, Riyadh, 11451 Saudi Arabia; 2 Botany and Microbiology Department, College of Science, King Saud University, PO Box 2455, Riyadh, 11451 Saudi Arabia

**Keywords:** *Rumex vesicarius*, Seed extracts, Fertility, Mice

## Abstract

*Rumex vesicarius* (RV) is an edible wild annual plant, and it is reported that it contains a good source of minerals, protein, and ascorbic acid. Several studies have indicated the anti-liver damage, anticancer, antimicrobial, and antioxidant properties of the RV plant. There are currently no reports regarding the effect of RV on fertility. Therefore, this study focuses on the impact of RV water seed extracts on mice fertility. RV plants were collected, and water seed extracts were prepared; 50 mg/kg body weight (BW) of this was then injected into the mice (male and female) using an oral feeding tube 5 days before mating (group I) or during caging of the females with the males for 1 week to detect their fertility rate. In the different female groups, no significant difference between their BW and their newborn’s BW in the treated and control groups was found. Female fertility, pregnancy, and offspring rates showed some variation within each female group and between the different female groups. In comparing the fertility and offspring rate between the different groups, there was a significant difference (*P* < 0.05) between groups I and III females and between groups I and IV females, while the other groups showed no significant differences. In contrast, the other groups showed no significant differences. Regarding the impact of the water seed extract on males, the BW was approximately the same in control and treated males.

## Introduction

*Rumex vesicarius* (RV) is an annual flowering plant in the family Polygonaceae with triangular to succulent leaves, and the flowers have conjoined seeds with white or pink transparent shutters. The flowering period is between January and March, and it reaches a length of 11–44 cm, width of 8 mm, and root length of 5–10 cm. It spreads from the West Asian continent to India, North Africa, and South Europe. It grown in dry temperate areas, and it has been cultivated in some wetlands in Tanzania and can grow in most soils, preferring wet and good drainage soils (Al-Rumaih et al. 2002 and Alfawaz (2006). Phytochemical analysis of RV shows that it contains several bioactive phytochemicals, such as flavonoids and anthraquinones; it is also a good source of minerals, protein, and ascorbic acid (Al-Rumaih et al. 2002; Alfawaz 2006; Abou Elfotoh et al. 2013). Since it is edible, the whole plant and its seeds were used in various ways, such as an aperitif and diuretic; it can also help in the treatment of anemia (Panduraju et al. 2009; Mostafa et al. 2011). In Saudi Arabia, RV is widely used as food, medicinal herb, and an antidote to scorpion and snake bites (Al-Yahya et al. 1990). There are several studies on the anti-liver damage (Tukappa et al. 2015; Ganaie et al. 2015), anticancer, antimicrobial, and antioxidant properties of RV (El-bakry et al. 2012; Fawzia et al. 2014; Tajdar et al. 2014; Laouini and Ouahrani 2017; Quradha et al. 2018) and on its use for treating diabetic rats (Reddy et al. 2017). *Rumex steudelli* (Tult) root extracts are used as an antifertility agent (Feroche 2015). Although some of the plant species are notorious and palatable to humans and animals, there remains insufficient knowledge regarding the effects of individual species on reproductive performance in animals (Zeitoun and Alsoqeer 2014). Other studies are conducted in vitro using RV seedlings (El-bakry et al. 2011; Nandini et al. 2013). According to the chemicals, content of the RV contains ascorbic acid, protein, lipids, tocoferol, and minerals. The RV also rich source of ß carotenes and anthraquinones particularly in roots such as emodin and chrysophanol, and vitamins (especially vitamin C), proteins, lipids and organic acids (Belanger et al. 2010) and other parts rumicine, lapathine (Nadkarni 1976) Lutein (Bhaskarachary et al. 2000) and some glycosides: vitexin, isovitexin, orientin and iso-orientin (Pullaiah and Ali Moulali 1997). Several studies reported that the RV is a good source of minerals such as K, Na, Ca, Mg, Fe, Mn, and Cu (Saleh et al. 1993; Al-Rumaih et al. 2002; Alfawaz 2006, and Filho et al. 2008, Ahmad et al. 2016). In addition, it has many important medicinal uses; the plant is stimulant, tonic, and acts as aphrodisiac agent (Gopal et al. 2008), the he high acidity flavour (Filho et al. 2008; Lakshmi et al. 2009). Gupta et al. (2008) analyzed ascorbic acid content in *R. vesicarius* L. In addition, Hariprasad and Ramakrishnan (2011) and Al-Mazroa et al. (2015) made a GC-Ms analysis of *Rumex vesicarius* L. Mouse was used for a long time as a model tool in reproductive physiology and medicine research as experimental models for human reproductive physiology (Handelsman and Walter 2020). Despite the importance of RV, there are no current reports regarding its effect on fertility. Therefore, this study focuses on the impact of RV water seed extracts on mice fertility.

## Materials and methods

Plant *Rumex vesicarius* (RV) samples were collected from wild of different border areas of eastern and southern Riyadh and from the borders of Wadi Hanifa at Riyadh area, between January and March 2020, without a necessary permissions for collection. The taxonomy of the RV plant was done by our co-author Dr Okla M. and confirmed by authenticated expert at the herbarium units taxonomist. A voucher specimen is deposited at the herbarium units at the Botany Department, College of Science, King Saud University.

### The water extraction from RV seeds

RV seeds are collected and dried at room temperature at 25–28°C for a month. Then, the seeds were ground to powder of approximately 100 g. This was added to a separation tube containing 1 L of distilled water for 48 h with repeated stirring; then, the solution was separated using a steam filter (Shahat *el al. *2015).

According to Reddy et al. (2017), to reach the desired concentration of water seed extracts (50 mg/kg), a 50-mL beaker was weighed and then filled with 5 mL of filtered fluid; it was then evaporated in an isotonic incubator (50–60°C for approximately 5 h). Afterward, the remaining extract was weighed, and then each mouse was injected with the desired dose, according to the following formula (Alhimaidi et al. 1998):

$$ \mathrm{Desired}\ \mathrm{dose}\ \left(50\ \mathrm{mg}/\mathrm{kg}\right)\times \mathrm{mouse}\ \mathrm{body}\ \mathrm{weight}\ \left(\mathrm{BW},\mathrm{g}\right)/\mathrm{RV}\ \mathrm{water}\ \mathrm{seed}\ \mathrm{extract}\ \mathrm{concentration}\ \left(\mathrm{mg}/\mathrm{mL}\right) $$

### Animal treatment

Eight- to 10-week-old adult female and male SWR mice (20–48 g) were obtained from the Animal House of the Zoology Department, College of Science, King Saud University. They were housed in a ventilated room at 25°C ± 2°C under a 12:12-h light/dark cycle. The animals were acclimatized at our lab for 2 weeks before the study and had free access to standard laboratory feed and water *ad libitum*.

### The experimental design

All animal treatments were performed according to the regulations and guidelines of the ethics committee at the King Saud University and the Institutional Animal Care at the King Saud University, CITI program, Reducing Pain and Distress in Laboratory Mice and Rats lab animals ID no. Record ID 31112900.

All animals used were weighed before and after the experiment. According to the treatment time either before or after mating with males, the females were divided into the following four groups:

#### Group I

Females treated with RV water seed extracts (50 mg/kg) for 5 days using an oral feeding tube served as the treated group (20 females), while those treated with water only served as the control group (20 females) 1 week before matting. Then, the females were caged with either five treated or five control males for 1 week to determine the effect of the water seed extracts on the mice fecundity. The females were checked daily in the morning for vaginal plug observation to confirm mating.

#### Group II

Females treated with RV water seed extracts (50 mg/kg) for 5 days using an oral feeding tube served as the treated group (20 females), while the control group (20 females) was treated with water only. They then were caged with five treated or five control males for 1 week to detect the effect of the water seed extract on the fertility of the females. They were also checked daily in the morning for vaginal plug observation to confirm copulation.

#### Group III

The females (*n* = 24) were caged with either five treated or five control males, and each female was checked daily in the morning for vaginal plug observation to confirm copulation. Then, 7 days post copulation, the pregnant females treated with RV water seed extracts (50 mg/kg) for 5 days using an oral feeding tube served as the treated group, while those treated with water served as the control group to detect the effect of the extracts on embryo implantation or pregnancy rate.

#### Group IV

The females (*n* = 24) were caged with five treated and five control males, and each female was checked daily in the morning for vaginal plug observation to confirm copulation. Then, to detect the effect of the extracts on embryo development, 10–12 days post copulation, the females were treated with the extract (50 mg/kg) for 5 days using an oral feeding tube, and they served as the treated group; others were treated with water only, and they served as the control group.

All matting data, offspring produced from each female group, and newborn mice weight were recorded.

### Ovary samples

At the end of the experiment, 5–6 females from each group were euthanized; then their ovaries were collected for histological examination. The ovary samples fixed in 10% neutral formalin were dehydrated and imbedded in paraffin wax and then sectioned and mounted in glass slides and stained with hematoxylin and eosin stain.

### Male groups

All male mice were weighed at the beginning and end of the experiment. They were divided into two groups: the first group contains 10 males treated (as the first group of females) with RV water extract (50 mg/kg), and the control group contains 10 males treated with distilled water using an oral feeding tube for 5 days. Each male were then caged with three females for 1 week; this is repeated with each female group. At the end of the experiment, males were euthanized; then sperm was collected from the epididymis in a phosphate buffer solution (PBS). Afterward, the testes was dissected and weighed. The sperm samples were analyzed using sperm microscopy (Hamilton–Thorne/IVOS) to calculate the sperm count, motility rate, and progressive motility.

At the end of the experiment, 10 males from the treated males (RV seeds water extract 50 mg/kg) and 10 control group were euthanized; then their tests were collected for histological examination. The testes samples fixed in 10% neutral formalin were dehydrated and imbedded in paraffin wax and then sectioned and mounted in glass slides and stained with hematoxylin and eosin stain.

### Statistical analysis

The mean BW of male, female, and newborn mice and the number of offspring produced were recorded and analyzed using the Mine Tab INSTAT program. Analysis of variance was performed using the methods of Kolmogorov and Smirnov. The fertility rates were tested using the Chi-square analysis (Barakat et al. 2018).

## Results

### The *Rumex vesicarius* impact on female mice fertility and pregnancy rates

The result of the different female groups (Tables 1 and 3) shows that there were no significant differences between the different females in their BW and their newborn’s BW in the treated and control groups (group I, 27.9 g; group II, 26.05 g; group III, 28.87 g; group IV, 25.82 g). The female fertility, pregnancy, and offspring rates showed some variation with each female group and between the different female groups. In group I, control females that mated with treated males showed a higher fertility rate (50% compared with the others with 40% and 20%); however, their offspring production rate was the lowest (5.6% compared with other females within the first group; 7.75–8 newborns per female). In group II, the fertility rate of the females varied between 30 and 40% per pregnant female; however, regarding the offspring rate, some variation occurred between the control females that mated with treated males, thus showing the lowest offspring rate (5.33%) compared with other females within the same group II (7–9.33 offspring per female). In group III, both treated and control females showed approximately the same pregnancy rates (50–66.6%). However, the offspring rate of the treated females had a lesser rate (5.3–5.5 newborn/female) than the control group III offspring rate (8–9 newborn/female). In group IV, the pregnancy rate of the treated females (66–83%) was higher than the control group IV females (36–60%) by approximately the same offspring rate in both treated and control groups IV females (4.16–6 newborn/female) (Table 1). Comparing the fertility rate using statistical analysis (Tukey–Kramer multiple comparison test) between the different groups, there was a significant difference (*P* < 0.05) between groups I and III females and between groups I and IV females, while the other groups showed no significant differences (Table 3).

**Table 1:** The mean data of female mice treated with *Rumex Vesicarius* seed extract compared to control group mated with treated or control males

Female group (no) mated with males	Female mean body weight and SEM+	Female fertility, pregnancy no, and %	Offspring no and rate	Newborn mean body wt. gm.
I-a Treated females (10) mated with treated males	26.8gm.+2.35	2/10=20%	17 8.5	1.56
I-b Treated females (10) mated with control males	28.4 gm.+1.70	4/10=40%	31 7.75	1.56
I-c Control females (10) mated with treated males	32.0 gm.+1.03	5/10=50%	28 5.6	1.65
I-d Control female (10) mated with control males	28.8 gm.+1.93	2/10=20%	16 8	1.52
II-a Treated females (10 ) with treated males	28.4 gm.+1.78	4/10=40%	33 8.25	1.59
II-b Treated females (10) mated with control males	25.6gm.+1.62	4/10=40%	28 7	1.55
II-c Control females (10) mated with treated males	25.2gm.+1.91	3/10=30%	28 9.33	1.40
II-d Control females (10) mated with control males	26.8gm.+1.61	3/10=30%	16 5.33	1.54
III-a Treated females (6) mated with treated males	28.66 gm.+1.60	3/6=50%	16 5.33	1.44
III-b Treated females (6) mated with control males	27.00gm.+1.91	4/6=66.6%	22 5,5	1.57
III-c Control females (6) mated with treated males	29.00gm.+1.69	4/6=66.6%	32 8	1.59
III-d Control females (6) mated with control males	30.00 gm.+1.86	4/6=66.6%	36 9	1.51
IV-a Treated females (6) mated with treated males	27.66gm.+2.09	5/6=83.3%	32 6.4	1.56
IV-b Treated females (9) mated with Control males	26.44gm.+1.90	6/9=66.6%	39 6.5	1.50
IV-c Control females (12) mated with treated males	30.50gm.+1.23	4/11=36.3%	24 6	1.47
IV-d Control females (12) mated with control males	27.60gm.+1.48	6/10=60%	25 4.16	1.43

### The histology of the ovary

**Fig. 1:**
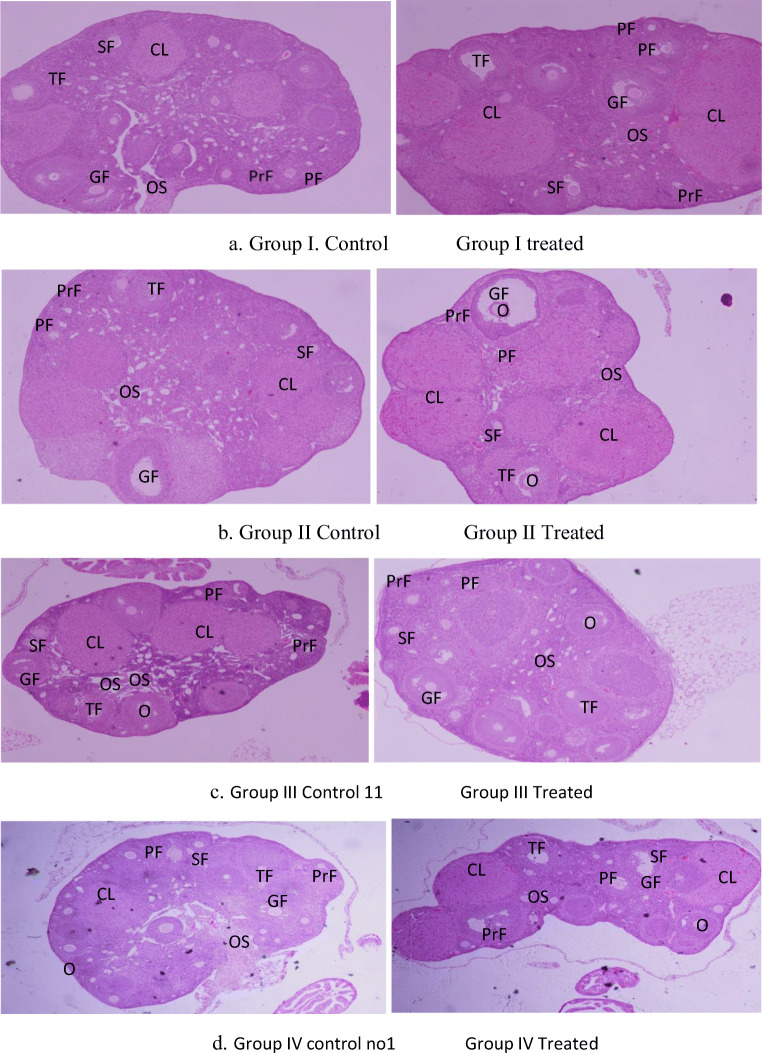
**a**, **b**, **c**, and **d** Section picture of mice ovaries treated with *Rumex Vesicarius* (RV) water seed extract before mating (group I), during mating (group II), and early pregnancy (group III) and late pregnancy (group IV) (10×10 magnification); PF, primordial follicle; PrF, primary follicle; SF, secondary follicle; TF, tertiary follicle; GF, Graafian follicle; CL, corpus luteum; O, oocytes; OS, ovary stoma

The histology of the ovary in Fig. 1a, b, c, and d illustrates the picture of the four different treated groups compared to the control groups either the 1st treated group I before 1 week of mating or during mating group II, while the third group III treated after 1-week post mating, and the fourth group treated during the second week post mating. The primordial follicles are oocytes surrounded by one layer of follicle cell and tend to be close to the ovary cortex, while the other follicles are surrounded by more than one layer of follicle cell, and they located from the cortex to the medulla of the ovary. The corpus luteum tends to be a large and prominent structure in the ovary (Fig 1a, b, and d). Tables 2 and 3 show the different follicle count rate per ovary (the primary, secondary, tertiary, Griffin follicle, and the corpus luteum per ovary). The number of each different follicles type per ovary vary from 1-4/ follicle type per ovary with no significant differences, except the Graafian follicle which it showed very low rate vary from (0.0 for treated group IV compared to the 1st and 2nd control group 0.42 Graafian follicle per ovary, which show a significant differences (p<0.05) (Table 3).

**Table 2:** The data of follicles and corpus lutetium counted in ovary of RV seeds water extract- treated mice

Groups	Ovary no	Primordial/ovary	Primary/ovary	Secondary/ovary	Tertiary/ovary	Graafian follicles/ovary	Corpus lutetium
Group I Control	12	31/12 (2.58)	40 (3.33)	18 (1.5)	37 (3.08)	5 (0.416)	46/12 (3.83)
Group I Treated	14	47/14 (3.35)	45 (2.14)	22 (1.57)	39 (2.78)	5 (0.35)	49/12 (3.5)
Group II Control	7	23/7 (3.385)	14 (2.0)	8 (1.15)	7 (1.0)	3 (0.428)	21/7 (3.0)
Group II Treated	6	7/6 (1.66)	9 ( 1.5)	9 (1.50)	14 (2.33)	2 (0.33)	28/6 (4.66)
Group III Control	9	14/9 (1.55)	25 (2.77)	24 (2.66)	30 (3.33)	2 (0.22)	36/9 (4.0)
Group III Treated	9	11/9 (1.22)	26 (2.88)	36 (4.0)	27 (3.0)	3 (0.33)	27/9 (3.0)
Group IV Control	9	12/9 (`1.33)	35 (3.88)	32 (3.55)	29 (3.22)	2 (0.22)	27/9 (3.0)
Group IV treated	6	12/6 (2.0)	14 (2.33)	9 (1.50)	15 (2.50)	0 (0)	20 (3.66)

**Table 3:** The multiple comparisons and statistical analysis in the fertility of the different female mice groups treated with RV seed water extract

Female group comparisons	Mean fertility rate differences	Fertility *P* value significance	Total mean Graafian follicle rate/ovary	Graafian follicle rate *P* value significance	Mean corpus luteum rate/ovary	Corpus luteum *P* value significance
Group I vs II	−0.250	*P*>0.05 ns	0.38/0.38	*P*>0.05 ns	3.65/3.76	*P*>0.05 ns
Group I vs III	−2.950	*P*<0.05 Sig	0.38/0.27	*P*>0.05 ns	3.65/03.5	*P*>0.05 ns
Group I vs IV	−2.875	*P*<0.05 Sig	0.27/0.13	*P*<0.05 Sig	3.50/3.1	*P*>0.05 ns
Group II vs III	−2.700	*P*>0.05 ns	0.38/0.27	*P*>0.05 ns	3.76/3.50	*P*>0.05 ns
Group II vs IV	−2.625	*P*>0.05 ns	0.38/0.13	*P*<0.05 Sig	3.76/3.10	*P*>0.05 ns
Group III vs IV	0.0750	*P*>0.05 ns	0.27/0.13	*P*<0.05 Sig	3.50/3.10	*P*>0.05 ns

### The *Rumex vesicarius* impact on male mice fertility

Table 4 shows the result of the impact of RV water seed extracts on the male mice mean BW, testes weight, sperm count, and motility rate. The mean BW of treated and control males was approximately the same (35.4 and 36.6 g, respectively). Regarding the right and left testes, the left testis was considered slightly higher in weight; however, the statistical analysis of the mean right and left testes weight of the treated and control male mice showed no significant differences. However, the Bartlett test suggests significant differences between the standard deviation (SD) of the treated male group (right testis weight, 0.0299; left testis weight, 0.02427) compared with the control group (right testis weight, 0.03538; left testis weight, 0.3204). The mean sperm count/mL of the treated males showed a higher sperm count (327.24–489.37 × 10^6^ sperm count/mL) compared with the control male group (281.16–283.16 × 10^6^ sperm count/mL). This variation was not statistically significant, but the Bartlett statistic test suggests that the difference between the SD is significant for the treated male group (234.14–365.84 sperm count/mL) compared with the control group (109.07–116.62 sperm count/mL) (Table 4). In addition, the total and progressive motility rates showed some variation between the different treated and control male groups, although not statistically significant (Table 4).

**Table 4:** Results of mice male mean body weight, test weight, sperm count, and motility rate of treated male mice with *Rumex vesicarius* seed water extract compared to control males

Male group and no	Mean body wt.gm.	Mean testes weight (wt.).gm. Right SEM+Left		Mean sperm count ×10^6^ Right SEM+Left		Mean motility rate Right SEM+Left		Mean progressive motility rate Right SEM+Left	
Treated males10	36.4+2.24	0.1127+0.0105	0.1080+0.0085	327.25+93.08	489.37+114.01	89.75+24.573	231.13+73.187	251.13+85.722	339.00+117.89
Control males10	35.4+1.60	0.1108+0.0111	0.2604+0.1013	281.16+44.528	283.16+47.61	39.00+105.29	131.67+36.89	252.5+ 87.38	140.83+147.5

### The histology of the tests

**Fig. 2:**
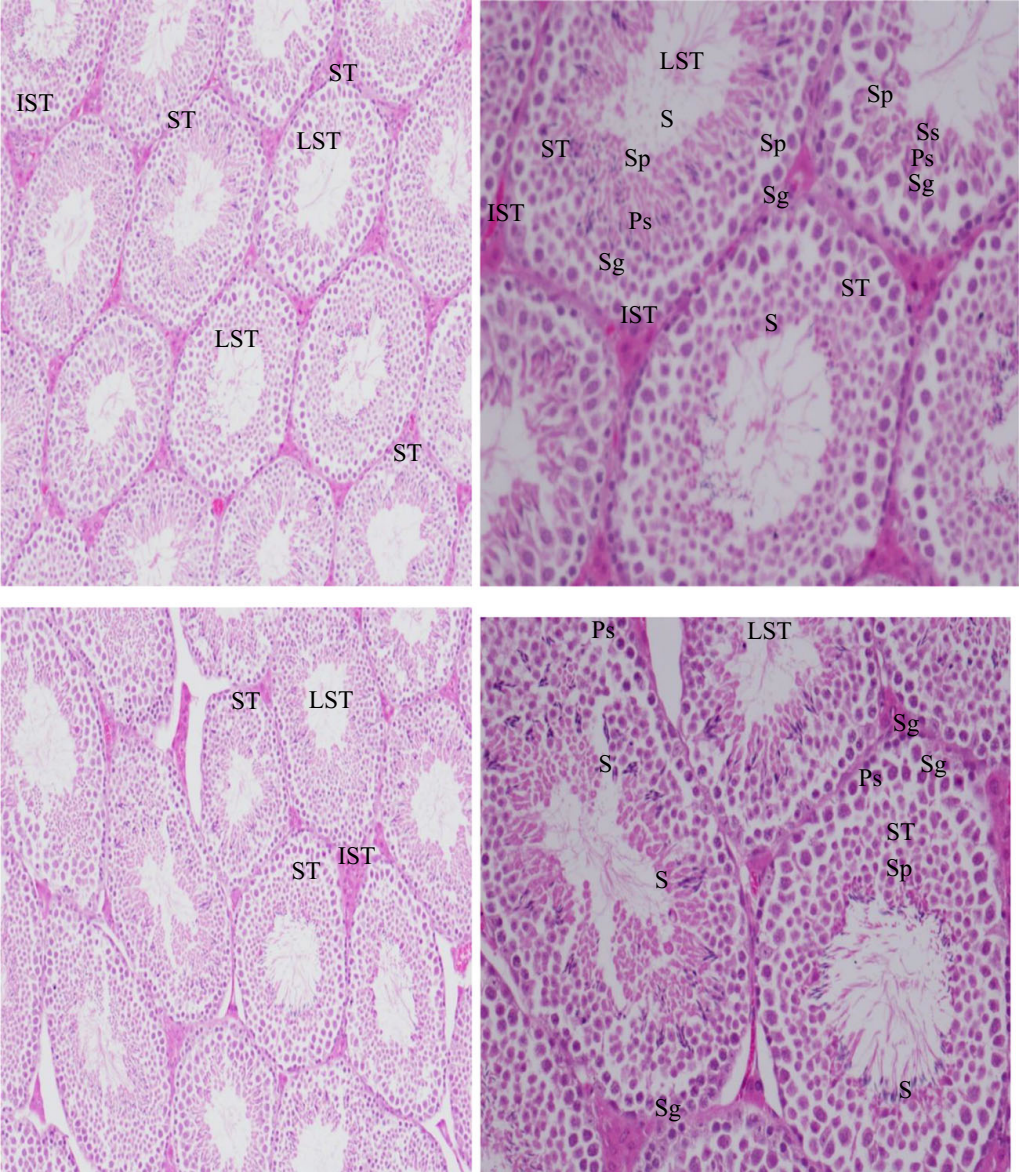
**a**, **b**, **c**, and **d** Transfer section (TS) picture of mice testes treated with *Rumex Vesicarius* (RV) water seed extract 50ul/gm./BWT (**a** and **b**), compared with the control male (**c** and **d**); ST, seminiferous tubules; IST, interstitial tissue; LST, lumen of the seminiferous tubules with different type of spermatogenesis of cell; Sg, spermatogonia; Ps, primary spermatocytes; Ss, secondary spermatocytes; Sp, sprematides; S, sperms. **a** and **b** T.S. mice tests treated with RV water seed extract (**a** 10×10 and **b** 10×20 magnification). **c** and **d** T.S. of control mice tests (**c** 10×10 and **d** 10×40 magnification)

The histology sections of the tests are illustrated in Fig. 2 (a, b treated, compared to the control group (c and d)). The treated group represents the same structure of the seminiferous tubes in structure with interstitial cells between the seminiferous tubules compared to the control group. The seminiferous tube contains the spermatogonia close to the basement membrane with dark stain and small size compared to the primary spermatocytes with light color which enters the 1st meiosis division to give the secondary spermatocytes. The spermatids resulted from the 2nd meiosis division of the secondary spermatocytes; they differentiate to result into sperm cells with tail. The sperms with dark stain of the head are close to the lumen of the seminiferous tubule lumens.

## Discussion

The calculation of the fertility rate of mice in our study was different from the regular fertility index of mouse from a mating trial, which is defined as the number of pups born per female per week during that trial or called Silver’s production efficiency index (Silver 1995). In addition, it is different from the study of Handelsma et al. (2020) where they calculate the fertility index as the slope of the regression of cumulative number of pups produced by a female over elapsed time in a monogamous mating trial by using a robust resampling technique. In our study, the female fertility rate based on counting the number of pregnant female and the total offspring sired in one time mating of each female groups divided in the total females in each group, although the results showed some statistical differences in the female fertility rate in the post pregnancy treatment groups III and IV, compared to the pre pregnancy treatment groups I and II. However, the offspring rate in groups II and I is higher than group III and IV. Our result of the offspring rate was better (5–8 in all different female groups) than the Lamb et al.’s (1985) study where their control pairs delivered an average of 4.75 l per fertile pair. Also the histology section of mice ovary showed the same results between the pre and post treatment group. Most of the post pregnancy treatment group (III and IV) compared to the pre pregnant-treated group (I and II) showed a large size of the corpus luteum with very low Graafian follicles; this is because most of the females were pregnant. From the results of this study, we concluded that RV water seed extracts have positive impact on mice reproduction and embryo development. This is confirmed by the histological comparison between the treated and control ovaries in different groups and follicle and corpus luteum not counted per ovary except the Graafian follicles as shown in Tables 2 and 3 and Fig. 1.

*Rumex vesicarius* (RV) showed no impact on the mean BW of males. Although the left testis of the control group showed higher weight compared to the treated male group, it also had a positive impact on the sperm count. This might agree with the study on *Moringa oleifera* leaf ethanolic extract supplementation at a level of 50 mg/kg BW effectively used to improve heat tolerance, oxidative status, and semen quality of rabbit bucks during summer (El-Desoky et al. 2017).

Therefore, the *Rumex vesicarius* (RV) seed extract is safe and effective for improving mice fertility compared with *Rumex steudelli* roots alcoholic extract at a dose of 200–400 mg/kg, which led to 95% abortion in rats (Feroche 2015). In addition, RV water seed extract supersedes the use of hydro-alcoholic fennel seed extracts on male rats at a dose of 35–280 mg/kg (Esrafil et al. 2016). This may be attributed to the use of these plant extracts at high doses and for long-term treatments. In addition, they are used as an antifertility agent, thus reducing the reproductively of rats. In addition, because of the varying contents of each plant part extract, different parts of the plant and its ripening stages might constitute an important factor to be considered during the evaluation of its impact.

## Conclusion

This is the first study that used the *Rumex vesicarius* (RV) seed water extracts, and its results showed safe and effective in improving mice fertility in male and female with their embryo development, which is indicated and supported in the histological section of the ovary and the tests of mice.

Therefore, the use of the *Rumex vesicarius* (RV) seed water extracts is safe and effective for improving mice fertility.

## Data Availability

All data of this manuscript are available upon request from the corresponding author**.**
